# Do osteoporotic fractures constitute a greater recalcitrant challenge for skeletal regeneration? Investigating the efficacy of BMP-7 and zoledronate treatment of diaphyseal fractures in an open fracture osteoporotic rat model

**DOI:** 10.1007/s00198-016-3771-8

**Published:** 2016-11-07

**Authors:** N. Mathavan, M. Tägil, H. Isaksson

**Affiliations:** 1 Department of Biomedical Engineering, Lund University, PO Box 118, 221 00 Lund, Sweden; 2 Department of Orthopaedics, Clinical Sciences, Lund University and Skåne University Hospital, Lund, Sweden; 3 Department of Applied Physics, University of Eastern Finland, Kuopio, Finland

**Keywords:** Bmp-7, Fracture healing, Osteoporosis, Ovariectomy, Zoledronate

## Abstract

**Summary:**

Osteoporotic fractures may pose a challenge for skeletal regeneration. This study investigates if pharmaceutical interventions such as bone morphogenetic protein 7 (BMP-7) alone or in combination with Zoledronate have equivalent efficacy in osteoporotic bone? Our findings suggest they do and that an osteoporotic bone environment may increase sensitivity to BMP-7.

**Introduction:**

Osteoporosis is thought to contribute to delayed or impaired bone healing. Bone morphogenetic protein 7 (BMP-7) alone or synergistically combined with zoledronate (ZA) has proven effective in augmenting the regenerative response in healthy young male rats. Yet their comparative efficacy in an osteoporotic bone environment is unknown. Our study aimed to answer the following questions using the ovariectomized (OVX) rat model of osteoporosis: Do osteoporotic fractures pose a greater challenge for skeletal regeneration? Are interventions with BMP-7-alone or combined with ZA of equivalent efficacy in osteoporotic bone?

**Methods:**

Sham operations (*n* = 33) or ovariectomies (*n* = 34) were performed in 12-week-old female Sprague-Dawley rats. Mid-diaphyseal open femoral osteotomies were created at 24 weeks of age and the rats allocated to either (i) untreated, (ii) BMP-7-only or (iii) BMP-7 + ZA treatment groups. At 6 weeks post-osteotomy, fracture healing was evaluated by radiography, μCT and 3-point bending mechanical tests.

**Results:**

Cumulatively, radiological, micro-structural and mechanical measures were equivalent in both healthy and osteoporotic environments. A reduced response to BMP-7-alone was observed in healthy rats that may be age/gender- or protocol/fracture-model dependent. Conversely, the BMP-7-only treated OVX group attained 100 % union in addition to significantly increased measures of mineralized bone volume, total callus volume, peak force and absorbed energy relative to untreated OVX fractures.

**Conclusions:**

Our findings refute the hypothesis that osteoporotic fractures constitute a greater recalcitrant challenge for skeletal regeneration. Furthermore, our results suggest that an oestrogen-deficient environment may in fact cause an increased sensitivity to BMP-7.

**Electronic supplementary material:**

The online version of this article (doi:10.1007/s00198-016-3771-8) contains supplementary material, which is available to authorized users.

## Introduction

Osteoporosis, a common metabolic bone disorder characterized by increased bone fragility, predisposes individuals to be more susceptible to fractures and is thought to contribute to recalcitrant fracture healing [1]. Much effort has been devoted to unravelling the aetiology of osteoporosis and, in particular, reversing the slide toward bone fragility through the preservation of bone mass. Surprisingly, less focus has been devoted to the potential impairment or diminution of the fracture healing process in the presence of osteoporosis [2].

Successful fracture repair is an orchestrated cascade of cellular and molecular events following a distinct temporal and spatial template, which culminates in the regeneration of bone, remodelling of the fracture site and restoration of function. Critical to the success of this skeletal regeneration process is the condition of the bone environment as characterized by its biological viability and biomechanical stability. Impairment in the form of age- or pathophysiology-related degradation and severity of trauma may lead to delayed or aborted osseous union, and sometimes necessitates interventions to specifically enhance the fracture healing process, in addition to stabilizing the fracture. Osteoporosis certainly impacts the severity and frequency of fractures but a definitive consensus is absent on its impact on the repair process. Some studies of ovariectomized (OVX) rats have reported an impaired healing process characterized by reduced callus bone formation, delayed or prolonged endochondral ossification and delayed callus remodelling [3–11]. Although frequently cited, these studies have shown inconsistent or contradictory results.

Moreover, evidence of osteoporosis-related anomalies at the molecular and cellular level have been reported that might have implications for the ability of bone to repair itself. Local gene expression studies have documented significantly lower expression levels of transcription factors critical to osteoblastogenesis such as runt-related transcription factor 2 (RUNX-2) and Osterix (Osx) and decreased expression of the bone formation marker Osteocalcin [12, 13]. Furthermore, the ratio of the expressions of the pro-osteoclastogenic factor RANKL to the anti-osteoclastogenic factor OPG was significantly higher in osteoporotic samples indicative of a diminished capacity in mediating osteoclastogenesis [12–14].

Clinically, there are too few studies or comparisons of fracture repair in healthy and osteoporotic individuals to substantiate the view that osteoporotic fractures constitute a recalcitrant challenge [2]. Certainly, fixation failure in osteoporotic fractures presents complications with significantly increased rates of non-unions/implant failure, post-operative mal-alignments and revision surgeries [15, 16]. Furthermore, the inverse correlation between bone mineral density and fixation strength or anchorage is well documented and thus failure or poor functional outcomes are not unexpected [17]. However, fixation failure is not equivalent to impaired fracture healing but is a consequence of poor bone quality and does not per se mean there is a decline in the bone regenerative capacity. Despite the absence of conclusive clinical evidence, it can be postulated that the physiological repair process is impaired or altered on the basis of the sparse literature that exists [12, 13, 18–22].

Fracture non-unions are rare, and although osteoporosis affects metaphyseal bone more than diaphyseal, non-unions are predominantly found in diaphyseal bones in the elderly osteoporotic population, like the femur or the humerus. In instances of fracture non-unions, the healing process could be augmented through the use of potent anabolic agents such as bone morphogenic protein 7 (BMP-7), alone or synergistically combined with anti-catabolic agents such as the bisphosphonate zoledronate (ZA) [23]. BMPs are powerful osteoinductive stimulators of new bone formation and are critical to the osteoregenerative process in promoting the terminal differentiation of committed osteoblastic precursors and osteoblasts from progenitor mesenchymal cells and osteoprogenitors [24, 25]. Multifunctional in nature, BMPs also modulate osteoclastogenesis and osteoclast homeostasis via the RANKL/RANK molecular mechanism or via direct stimulation of osteoclastic activity [23]. Consequently, the potential for prematurely induced bone and callus resorption has been documented [26]. To counter the upregulated catabolic response, this has necessitated the use of antiresorptives in conjunction with BMPs to optimize the anabolic potency of BMPs. Antiresorptive bisphosphonates such as ZA are primarily used in the treatment of osteoporosis for preservation of bone mass through promotion of osteoclast apoptosis [27].

Pharmacological interventions such as these should be based on the understanding of the bone environment and the influence of the environment on treatment efficacy. Use of BMP-7 alone or in combination with ZA has proven to substantially increase callus formation and enhance fracture healing in healthy young male rats [23, 28, 29]. Yet their efficacy in an osteoporotic bone environment or in older female rats remains unclear [30]. The consequences of an imbalance that favours bone resorption in an osteoporotic bone environment compounded with the upregulated catabolism of BMP-7 may portend as-yet-unknown complications to the fracture healing process. Therefore, the current study aimed to answer the following questions in a recalcitrant open-fracture model: Does the diminished biological viability of the osteoporotic bone environment pose a greater challenge for skeletal regeneration? And if so, are interventions with BMP-7 alone or in combination with ZA of equivalent efficacy in osteoporotic bone?

## Materials and methods

### Experimental model

Female Sprague-Dawley rats (Charles River, Germany) at 12 weeks of age either underwent ovariectomy (*n* = 34) or were sham-operated (*n* = 33). Using this model, we have previously established the presence of extensive structural deterioration characteristic of ovariectomy-induced osteoporosis at multiple anatomical sites in the 18 weeks following ovariectomy [31]. Consequently, at 24 weeks of age, mid-diaphyseal osteotomies were created in the right femur of all rats in accordance with a previously described open fracture model of recalcitrant non-unions [29, 32]. This model leads to approximately 50 % non-unions at the 6-week time point in young healthy male rats [29, 32]. The rats were randomly allocated to one of six treatment groups: (i) Control (untreated), (ii) OVX (untreated), (iii) Control (BMP-7 only), (iv) OVX (BMP-7 only), (v) Control (BMP-7 + ZA) and (vi) OVX (BMP-7 + ZA). Animals were maintained on ad libitum access to food and water, housed in pairs and permitted unrestricted weight bearing. In line with the fracture model protocol, the rats were euthanized at 6 weeks post-osteotomy. Approval of laboratory animal care and experimental protocol was obtained from the institutional animal ethics and scientific advisory committee (Ethical Permission No. M 316-11).

### Surgery and drug administration

A putty was made by mixing 2 mg of BMP-7 in 570 mg bovine collagen (Osigraft, Stryker Biotech, Malmö, Sweden) with 200 mg carboxymethyl cellulose (CMC) and apportioned in 50 μg doses [23]. Anaesthesia was induced through intraperitoneal injection of a solution constituting saline, diazepam (2.5 mg/mL) and pentobarbital-natrium (15 mg/mL). Transverse femoral osteotomies at the mid-diaphysis were created using an oscillating power saw and the fractures were stabilized and fixed with an intramedullary K-wire [32]. Treatments were subsequently administered based on group allocation. The putty was placed circumferentially around the osteotomy and the muscle fascia closed by running sutures to contain the putty before the skin was closed. At 2 weeks following surgery, the rats received a single subcutaneous injection of either saline or ZA (0.1 mg/kg) (Zometa, Novartis, North Ryde, NSW, Australia). The 0.1 mg/kg dosage of ZA administered is approximately equivalent on a milligramme to kilogramme basis to a once yearly dose received by humans for the treatment of osteoporosis. At 6 weeks post-osteotomy (30 weeks of age), the rats were sacrificed by an intraperitoneal injection of pentobarbital sodium. Body weight at time point of sacrifice was 314 ± 29 g and 420 ± 36 g for sham-operated and ovariectomized rats, respectively (*p* < 0.001). Postmortem, the osteotomized femurs and the intact contralateral femurs were harvested and all K-wires extracted.

### Radiographs

Antero-posterior radiographs of all femurs were obtained using a clinical X-ray system (GE Healthcare Discovery, Fairfield, CT, USA). Degree of fracture union was visually assessed blinded by an orthopaedic surgeon. It was classified as either demonstrating (1) complete healing (remodelled fracture gap with callus bridging both medially and laterally and no visible fracture line), (2) complete union (callus bridging both medially and laterally but with visible fracture line), (3) partial union (callus bridging either medially or laterally) or (4) non-union.

### Quantitative micro-computed tomography

Fracture sites of the femurs were imaged with Skyscan 1172 microCT system (v. 1.5, Skyscan, Aarteselaar, Belgium), using an isotropic voxel size of 15 μm. Tomographic image acquisition was conducted with X-ray source settings of 100 kV/100 μA, 8–10 repeated scans and the use of a 0.5 mm aluminium filter. Image reconstruction (NRecon Skyscan, v 1.5.1.4) included corrections for ring artefacts and beam hardening.

The volume enclosed by a proximal and distal distance of ±1.5 mm from the fracture line was defined as the boundaries of the region of interest (ROI). Analysis of ROIs consisted of quantification of the total callus volume, highly mineralized bone volume, bone volume fraction (BV/TV) inside the callus and average tissue mineral density using custom morphometric scripts written in MATLAB (v 8.50 (R2015a) Mathworks, Massachusetts, USA). Calibration phantoms of calcium hydroxyapatite with densities of 0.25 and 0.75 g/cm^3^ and a water phantom were scanned and reconstructed in accordance with the manufacturers’ protocol. Highly mineralized tissue corresponded to bone mineral densities greater than a threshold of 0.642 g/cm^3^ and poorly mineralized tissue was defined as densities in the threshold range between 0.410 and 0.642 g/cm^3^ [33]. Grayscale values corresponding to these thresholds were identified based on the calibrated reference densities.

### 3-Point bending tests

All fractured femurs and corresponding contralateral femurs were tested in 3-point bending (Instron 8511 load frame, High Wycombe, UK/MTS TestStar II controller, Minneapolis, USA). With the fracture line of the callus positioned directly under the central loading point, the femurs were subject to a preload of 10 N at a rate of 0.1 mm/s, allowed to adapt for 10 s whereupon they were loaded until failure at a constant rate of 1.0 mm/s. Peak force, extrinsic stiffness and absorbed energy were calculated from the recorded load-displacement curves.

### Statistics

Statistical analysis was performed using the Mann-Whitney *U* test for testing significant differences between Control and OVX groups and between treatment groups for all measurement parameters (SPSS, v22, SPSS Inc.). Differences in mechanical properties between fracture and contralateral control femurs were tested using the Wilcoxon signed rank test.

## Results

### Radiographs

Radiological healing rates were similar between Control and OVX groups across all treatment groups (Table 1). In untreated rats, more than half of fractures resulted in non-union with healing rates of 43 and 44 % observed in Control and OVX rats, respectively. In three of the four treatment groups which included BMP-7, radiological healing was observed in all (100 %) rats whereas with the BMP-7 only treated Control group a 70 % success rate was observed.

**Table 1:** Radiographic scoring of healing at 6 weeks post-fracture based on radiographs of the fracture site

		All Unions			All unions (1) + (2) + (3)	(4) Non-union
Group	*N*	(1) Union (remodelled fracture gap)	(2) Union (bridged, visible fracture line)	(3) Partial union (either medial or lateral bridging)		
Untreated Control	14	2/14	3/14	1/14	6/14 (43 %)	8/14 (57 %)
Untreated OVX	16	–	5/16	2/16	7/16 (44 %)	9/16 (56 %)
BMP Control	10	–	4/10	3/10	7/10 (70 %)	3/10 (30 %)
BMP OVX	10	5/10	5/10	–	10/10 (100 %)	–
BMP + ZA Control	9	6/9	1/9	2/9	9/9 (100 %)	–
BMP + ZA OVX	8	3/8	5/8	–	8/8 (100 %)	–

### Quantitative micro-computed tomography

**Fig. 1:**
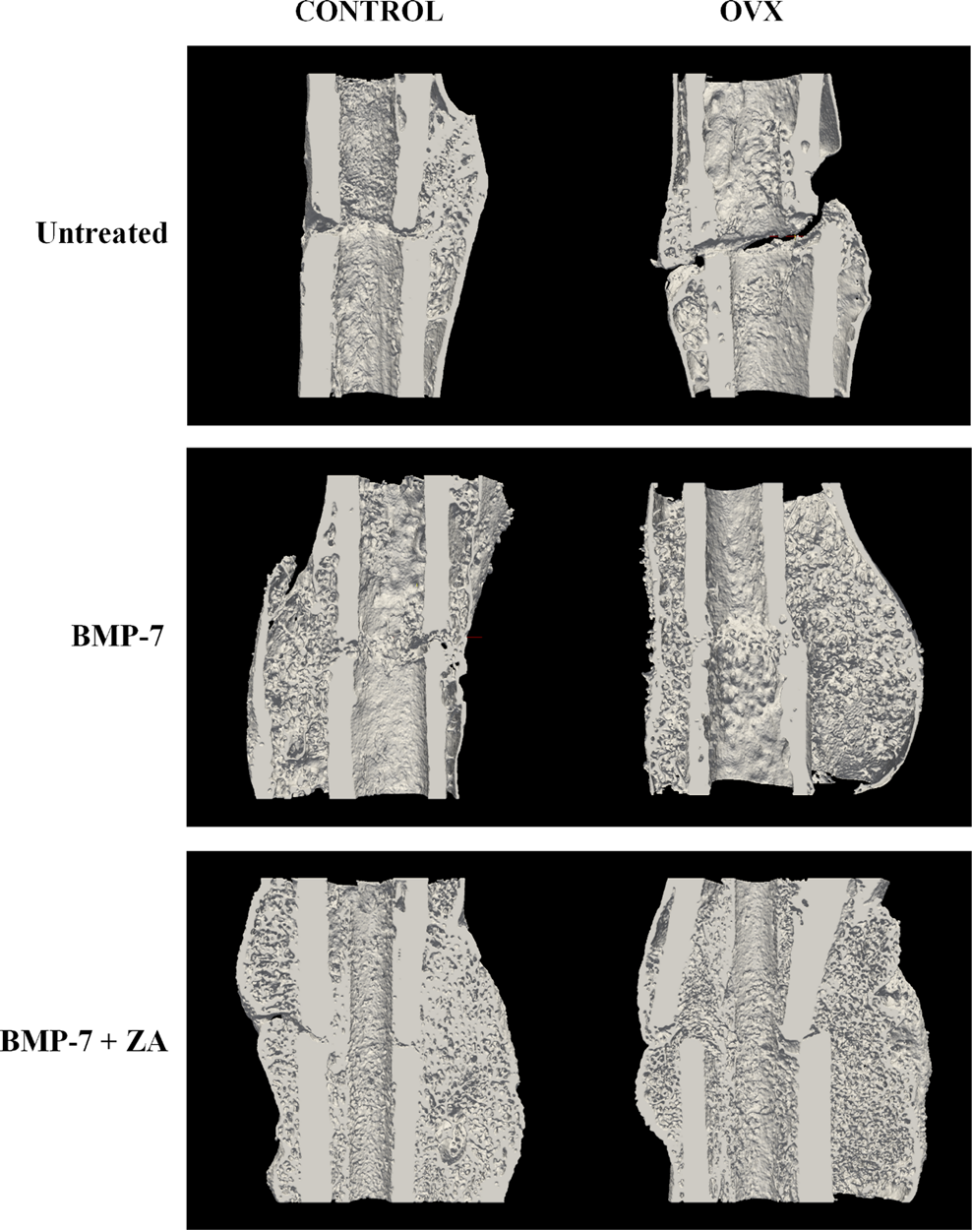
MicroCT reconstruction of the cross-section of the fracture site for a representative sample based on the median total callus volume sample from each group to illustrate the influence of the treatments on callus microarchitecture (ParaView, v3.1, Kitware Inc.)

Total callus volume was significantly greater in BMP-only treated OVX rats compared to BMP-only treated Control (non-OVX) rats (*p* < 0.001) (Table 2, Fig. 1). However, no significant differences in mineralized bone volumes were present in comparisons of Control and OVX rats irrespective of treatment. In BMP-only and BMP + ZA treated groups, bone volume fraction (BV/TV) was significantly lower in OVX rats than Control rats (*p* < 0.01, *p* < 0.001). Other noteworthy distinctions between Control and OVX rats include a significantly lower average tissue mineral density in the latter in the BMP-only treatment groups (*p* < 0.05).

**Table 2:** Micro-structural parameters based on micro-computed tomographic images and mechanical three-point bending data. Statistical comparison based on Mann-Whitney U-test between Control and OVX groups

		Untreated		p-value	BMP-7		p-value	BMP-7 + ZO		p-value
		Control	OVX		Control	OVX		Control	OVX	
		*(N = 14)*	*(N = 16)*		*(N = 10)*	*(N = 10)*		*(N = 9)*	*(N = 8)*	
Total Callus Volume (mm^3^)	Mean	54.1	57.3		63.5	122.1	***	101	126.5	
	SD	15.4	12.3		19.0	24.3		29.2	29.6	
High Mineralized Bone Volume (mm^3^)	Mean	27.8	25.5		33.7	34.6		61.5	62.8	
	SD	14.0	9.4		6.4	5.2		15.0	18.2	
Low Mineralized Bone Volume (mm^3^)	Mean	7.5	6.9		5.7	6.5		14.3	17.7	
	SD	3.2	1.7		1.7	1.6		6.6	6.8	
Bone Volume Fraction (%)	Mean	49.6	44.0		54.8	28.9	***	61.8	49.6	**
	SD	16.2	12.8		8.5	4.4		5.2	7.7	
Tissue Mineral Density (g/cm^3^)	Mean	0.99	1.01		1.20	1.15	*	1.08	1.05	
	SD	0.22	0.20		0.06	0.05		0.11	0.04	
		*(N = 6)*	*(N = 7)*		*(N = 7)*	*(N = 10)*		*(N = 9)*	*(N = 8)*	
Peak Force (N)	Mean	83.1	60.5		80.8	96.1		178	177	
	SD	32.6	20.9		28.4	26.0		62.2	95.0	
Extrinsic Stiffness (N/mm)	Mean	175.5	74.0		108.0	97.3		239	160	
	SD	90.1	71.3		64.1	53.9		123	63.2	
Absorbed Energy (N mm)	Mean	18.8	26.5		31.1	54.5		87.1	138	
	SD	9.9	13.8		11.9	24.3		40.1	120

Furthermore, in comparisons between treatments, mineralized bone volumes were significantly augmented in the BMP + ZA groups compared to all other treatment groups irrespective of bone condition (*p* < 0.01; *p* < 0.001) (Table 3, Fig. 1). In measures of total callus volume, the response to treatments differed between Control and OVX rats. Callus volumes were larger in BMP + ZA treated Control rats relative to all other treatment groups (*p* < 0.01; *p* < 0.001). In contrast, both BMP-only and BMP + ZA treated OVX rats exhibited increases in callus volumes relative to untreated rats (*p* < 0.001). Comparisons of callus cross-sections in BMP-only treated femurs revealed distinctly different structures in Control and OVX rats. Notably, the larger calluses in OVX rats were characterized by thin callus walls enclosing sparse trabecular architecture (Supplementary material). This is reflected in the BV/TV parameter which is significantly lower in BMP-only treated OVX fractures compared with untreated or BMP + ZA treated OVX fractures (*p* < 0.01; *p* < 0.001).

**Table 3:** Micro-structural parameters based on micro-computed tomographic images and mechanical three-point bending data. Statistical comparison based on Mann-Whitney *U* test between treatment groups

		Control			OVX		
		Untreated	BMP	BMP + ZA	Untreated	BMP	BMP + ZA
Total callus volume (mm^3^)	Mean	54.1	63.5	101	57.3	122.1	126.5
	SD	15.4	19.0	29.2	12.3	24.3	29.6
	*p* value			*** ^oo^		***	***
High mineralized bone volume (mm^3^)	Mean	27.8	33.7	61.5	25.5	34.6	62.8
	SD	14.0	6.4	15.0	9.4	5.2	18.2
	*p* value			*** ^ooo^		*	*** ^ooo^
Low mineralized bone volume (mm^3^)	Mean	7.5	5.7	14.3	6.9	6.5	17.7
	SD	3.2	1.7	6.6	1.7	1.6	6.8
	*p* value			** ^oo^			*** ^oo^
BV/TV (%)	Mean	49.6	54.8	61.8	44.0	28.9	49.6
	SD	16.2	8.51	5.23	12.8	4.44	7.67
	*p* value			^o^		**	^ooo^
Tissue mineral density (g/cm^3^)	Mean	0.99	1.20	1.08	1.01	1.15	1.05
	SD	0.22	0.06	0.11	0.20	0.05	0.04
	*p* value		*	^oo^			^oo^
Peak force (N)	Mean	83.1	80.8	178	60.5	96.1	177
	SD	32.6	28.4	62.2	20.9	26.0	95.0
	*p* value			** ^oo^		*	** ^o^
Extrinsic stiffness (N/mm)	Mean	175.5	108	239	74.0	97.3	160
	SD	90.1	64.1	123	71.3	53.9	63.2
	*p* value			^o^			* ^o^
Absorbed energy (N mm)	Mean	18.8	31.1	87.1	26.5	54.5	138
	SD	9.9	11.9	40.1	13.8	24.3	120
	*p* value			*** ^ooo^		*	** ^o^

* *p* < 0.05; ** *p* < 0.01; *** *p* < 0.001 when compared to untreated femurs and ^o^ *p* < 0.05, ^oo^ *p* < 0.01, ^ooo^ *p* < 0.001 when compared to BMP only treated femurs

**Fig. 2:**
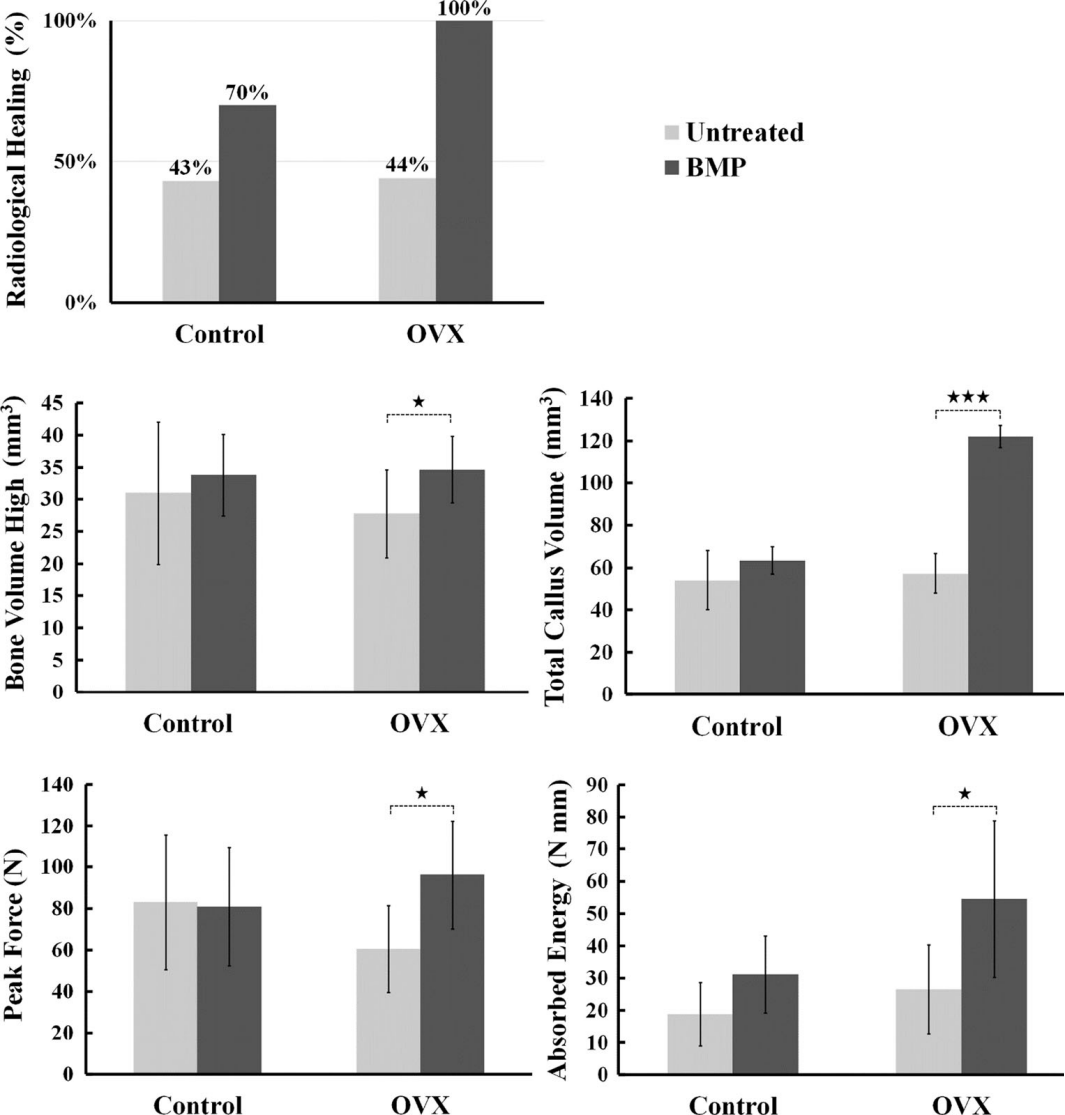
Radiological union rates, microstructural parameters based on micro-computed tomographic images and mechanical three-point bending data of healthy and osteoporotic fractures treated with BMP-7 alone. Statistical comparison based on Mann-Whitney *U* test of the osteogenic response of healthy and osteoporotic fractures to treatment with BMP-7 alone. (*p < 0.05; ***p < 0.001)

Noteworthy also, were the increased presence of significant differences between OVX treatment groups than between Control treatment groups (Table 3). In comparisons of untreated and BMP-treated OVX rats, significantly higher measures of total callus volume (*p* < 0.001) and highly mineralized bone volume (*p* < 0.05) were observed in the latter group but these differences were notably absent in the corresponding Control groups (Table 3, Fig. 2).

### 3-Point bending tests

**Fig. 3:**
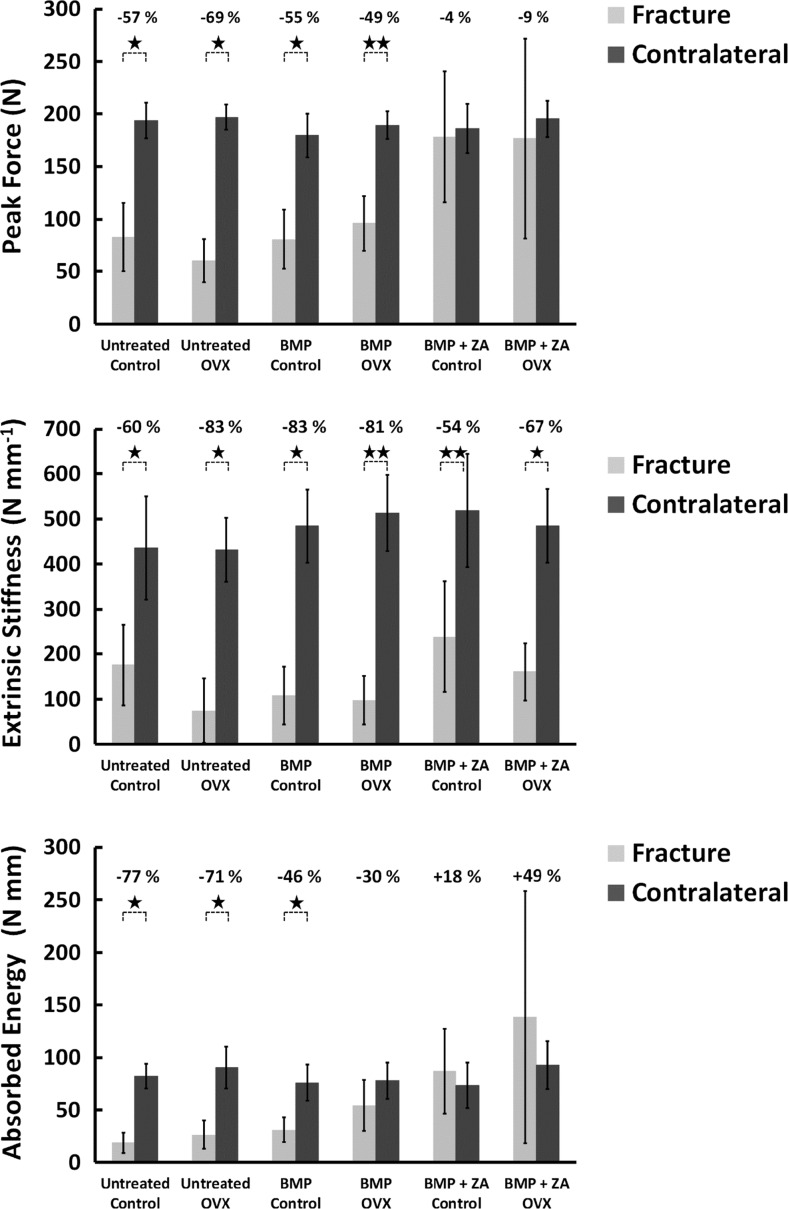
Mechanical three-point bending tests of the contralateral femurs. Percentage differences and the statistical comparison based on the Wilcoxon signed rank test between the fracture femurs and the contralateral femurs are presented (* *p* < 0.05; **p < 0.01)

Non-unions were excluded in the analysis of mechanical parameters. No significant differences were noted in the mechanical properties of Control and OVX groups for all treatments (Table 2). Across treatment groups, BMP + ZA outperformed all others groups in terms of peak force, extrinsic stiffness and absorbed energy (Table 3). Furthermore, BMP + ZA generated peak force and absorbed energy values that were similar to the values obtained for the contralateral control femurs (Fig. 3). In comparison, untreated fractures and BMP-treated fractures recorded significantly diminished peak force and extrinsic stiffness values relative to the corresponding contralateral femurs (*p* < 0.05; *p* < 0.01). Absorbed energy values between fracture and non-fracture femurs were also significantly lower in the untreated groups and the BMP-treated Control group (*p* < 0.05) but the difference was not of statistical significance in the BMP-treated OVX group.

## Discussion

The objective of the present study was to analyse the regenerative capacity of osteoporotic bone and to assess the efficacy of pharmaceutical interventions in enhancing this capacity using an established ovariectomized rat model of osteoporosis. Literature on osteoporotic fracture repair is contradictory with publications suggesting either impairment at the early stages of fracture healing, impairment at the late stages of fracture healing or no impairment of the healing process. Our findings demonstrate the fracture healing capacity in an oestrogen-deficient environment to be equivalent to that in healthy bone in all three treatment groups in a long bone model of recalcitrant healing.

### Fractures in ovariectomized rats are no more recalcitrant than fractures in non-ovariectomized rats

Cumulatively, our findings suggest that at 6 weeks post-osteotomy, OVX fractures appear to be no more recalcitrant than equivalent healthy fractures. The efficacy of the treatments is retained in the osteoporotic bone environment, if not surpassed in the case of BMP-7 only. Across radiological, micro-structural and mechanical measures of bone quality, the impaired biological viability and biomechanical stability of OVX-induced osteoporotic bone does not appear to adversely impair the fracture repair process.

The literature, which is mainly limited to OVX rat studies, is far from conclusive with findings both supporting and contradicting the hypothesis that osteoporotic fractures constitute a greater recalcitrant challenge. Namkung-Matthai et al. used an open fracture model and reported diminished measures of callus cross-sectional area, bone mineral density (BMD) and biomechanical properties at 3 weeks post-osteotomy relative to healthy controls [3]. Decreased callus widths and callus areas at early fracture healing time points and a reversed trend of increased callus areas at late time points were reported by both Shi et al. and Hao et al. [4, 5]. Their findings are suggestive of a healing process characterized by reduced bone formation and delayed callus remodelling. Hao et al. documented inferior microarchitecture parameters and callus bone mineral density across weeks 4, 8 and 12 following osteotomy and diminished biomechanical properties at week 12 [5]. Furthermore, the results of Chung et al. supported the notion of delayed callus remodelling based on their measures of the ratio of the bone remodelling markers osteoprotegrin (OPG) and RANKL which was lower at weeks 4 and 8 in OVX rats [6]. The findings of Islam et al. are also suggestive of a potential prolongation in the healing process with the authors noting a sustained endochondral ossification phase characterized by elevated expression of BMP-2 and TNF-α and increased numbers of predominantly osteoclasts but also osteoblasts and TNF-α + cells in OVX rats [7]. Together, these studies suggest a decline in the capacity for fracture repair in osteoporotic fractures, characterized by decreased callus formation, delayed or prolonged endochondral ossification and delayed or diminished callus remodelling.

Conversely, the literature also contains publications, remarkably overlooked, that document no differences or only marginal differences in the healing processes of healthy and OVX rats. Using an open fracture model, Cao et al. examined healing at 6 weeks and 16 weeks following fracture induction and observed no effects of osteoporosis in their evaluations of bone callus area, bone mineral content (BMC), moment of inertia, biomechanical properties and intrinsic material properties at either time point [34]. Likewise, Wheeler et al. reported no differences in peak bending force and stiffness in comparison of OVX and control rats at weeks 4, 6 and 8 following fracture [35]. Kubo et al., also using an open fracture model, found no differences at 6 weeks post-osteotomy and reported decreases in BMD and histological distinctions but comparable mechanical properties at 12 weeks post-osteotomy [36]. Notably, callus widths did not significantly differ at 6 or 12 weeks. Shi et al. observed only marginal discrepancies in microarchitecture parameters at weeks 2, 4 and 8 and no differences in mechanical properties at week 8 [4]. Similarly, McCann et al. documented no significant differences in radiological healing at weeks 1, 2 and 4 in an open fracture model [11]. And although the authors reported radiological healing to differ at weeks 6 and 8, this was not evident in their measures of mechanical strength at weeks 6 and 8.

Of relevance in the interpretation of the findings in these studies is the significance of age, anatomical site and time post-ovariectomy in determining the extent of osteoporotic deterioration of the bone environment. And consequently, comparisons between studies are somewhat problematic. In general, studies which reported differences in healing featured animals that were older at ovariectomy. Neither the time between ovariectomy and fracture creation nor the type of fracture model (i.e. open or closed) appeared to clearly influence the outcome. Nevertheless, collectively, the published pre-clinical studies and clinical studies have yet to formulate a compelling and conclusive argument for the recalcitrant nature of osteoporotic fractures. And our results are in agreement with this.

### Does an oestrogen-deficient environment increase sensitivity to BMP-7?

Our findings indicate that the osteogenic response with BMP-only treatment is different in healthy and osteoporotic bone environments. BMP-only treated OVX fractures exhibited larger calluses equivalent in volume to those achieved with BMP + ZA (Table 3). Notably mineralized bone volumes were unaffected since the calluses were characterized by thin walls and sparse trabecular architecture (Supplementary material). This was not necessarily detrimental to healing, since no significant differences were observed in the mechanical parameters between BMP-treated Control and OVX groups (Table 2). OVX-induced osteoporosis is characterized by high bone turnover and it can be speculated that the large callus volumes are a product of the upregulated anabolism of OVX coupled with the potent anabolic effect of BMP-7. Similarly, the augmented catabolic consequences of OVX and BMP-7 yield the sparse microarchitecture within the calluses.

Noteworthy also was the response in healthy rats where BMP-7 alone was not as beneficial as expected when compared to untreated fractures. Based on radiographic healing, untreated fractures attained a union rate of 43 % and BMP-7 treated fractures attained a union rate of 70 %. Indeed, across microstructural and mechanical measures, treatment with BMP-7 alone did not prove to be of much benefit when compared to untreated control rats. Conversely, the OVX group achieved 100 % radiological union in addition to significantly increased measures of mineralized bone volume, total callus volume, peak force and absorbed energy relative to their untreated OVX counterparts (Fig. 2).

It should be noted that comparisons of union rates between groups in this study are not sufficiently powered to detect significant differences. However, the fact that BMP-7 alone did not achieve 100 % radiological union in Control rats is surprising since our previous studies of BMP-7 in combination with bone autograft or allograft in young male rats achieved 100 % radiological unions at 6 weeks [28, 29] and a segmental defect model using BMP-7 alone achieved a similar result at 8 weeks [23]. Moreover, using the same fracture model in 7–8 week old healthy male rats, a 100 % union rate was also observed with BMP-7 at 6 weeks in one of our ongoing, unpublished studies.

Of relevance when interpreting the findings of fracture healing studies is the predisposition to often use younger, male animals compared to the current study. Much of the pre-clinical studies on the osteoinductive potency of recombinant BMP-7 were demonstrated in rabbit, canine, ovine and non-human primate models of diaphyseal critical-sized segmental defects [37–40]. Furthermore, these findings have been corroborated by clinical studies that have produced comparable results to autograft based on assessments of radiological healing and functional outcomes [38, 41]. To the best of our knowledge, the age or gender dependent efficacy of BMP-7 is yet to be investigated. Gender discrepancies in the physiological and pathophysiological mechanisms underlying ageing and osteoporosis exist. For instance, Föger-Samwald et al. reported no significant differences in local gene expression of the osteoclast related genes RANKL and OPG between elderly men with age-related osteoporosis and elderly men with osteoarthritis [42]. In a follow-up study, the authors reported a significantly increased ratio of RANKL to OPG in elderly osteoporotic women relative to age-matched control cohort of elderly women [13]. Thus, the age or gender dependency of BMP-7 is a valid question.

Unlike the segmental defect models, non-union models pose a different challenge as the potential for spontaneous healing is present and the role of BMPs is to augment this response. In this study, this was not the response in healthy rats. A review of the literature was not able to uncover studies of the effectiveness of BMP-7 in a non-union model in healthy rats that is independent of other osteo-stimulatory agents such as grafts or scaffolds. Conversely, if indeed there is perturbation in osteoporotic bone of the processes governing the fracture healing process (which is not necessarily inhibitory), our findings suggest that the presence of BMP-7 is significantly beneficial. There is some evidence to suggest the involvement of BMPs in the pathogenesis of osteoporosis [43]. And if so, it could be asserted that when the spontaneous healing process is fully functional, the effect of BMP-7 is diminished.

Nevertheless, the sensitivity of BMP-7 in healthy and osteoporotic older female rats remains to be clarified and it is undoubtedly valid to question if our findings are an age/gender-dependent characteristic or strictly a model-related or protocol-related consequence. Moreover, BMP-7 induced bone formation is indeed pleiotropic in nature with a susceptibility to fracture site variation in terms of the local mechanical environment, pH, extent of vascularization, size of the hematoma, release kinetics, surgical technique and local population of connective tissue progenitor cells [44]. Thus further investigation is warranted.

### BMP + ZA is by far the most effective treatment but potentially longer recovery due to delayed remodelling is expected

Finally, the effectiveness of BMP-7 + ZA irrespective of whether the underlying bone was healthy or osteoporotic further validates adoption of the anabolic—anti-catabolic paradigm in optimizing pharmacological interventions. Complete union in all samples was observed at 6 weeks, in addition to dramatically increased callus and mineralized bone volumes and the mechanical strength parameters of peak force and absorbed energy attaining values equivalent to that of the contralateral femurs. However, this is expected to be potentially offset by delayed remodelling of the callus and delayed callus maturation [45]. And with regard to functional outcomes, it may prove to be problematic if the substantial callus formed is present for an extended length of time.

### Limitations

The current study is not without its limitations, most notably the presentation of results at a single time point. The use of a single time point at 6 weeks post-osteotomy was justified in the context of the number of groups involved and that it allows comparisons of outcomes with our previous publications reporting results from the non-union model at the same time point. In addition, the analysis of mechanical properties does not account for the disparity in body weight between Control and OVX groups (100 g difference). Finally, the choice of a diaphyseal model to study osteoporotic fractures could be questioned with the majority of the fractures being metaphyseal. However, the aim of the study was primarily to test whether BMP can be used to treat or prevent diaphyseal non-unions as effectively in older osteoporotic animals as previously shown in younger animals.

## Conclusion

Our findings refute the hypothesis that OVX fractures constitute a greater recalcitrant challenge for skeletal regeneration. Treatment with BMP-7 alone or synergistically with ZA was of equivalent efficacy or better in an osteoporotic bone environment. Contrary to expectations, the response to BMP-7 alone was limited in healthy rats. Earlier pre-clinical fracture healing studies of BMP-7 have predominantly used younger, male animals and segmental defect models. Thus a potential age/gender or fracture model dependency cannot be excluded. Conversely, the augmented response to BMP-7 in OVX fractures suggests that the oestrogen-deprived bone environment may in fact increase sensitivity to BMP-7. Finally, our results using BMP-7 + ZA further validates adoption of the anabolic—anti-catabolic paradigm in optimizing pharmacological interventions of the regenerative process.

## Electronic supplementary material

ESM 1 (PDF 946 kb)
