# Supplementary material for: Do osteoporotic fractures constitute a greater recalcitrant challenge for skeletal regeneration? Investigating the efficacy of BMP-7 and zoledronate treatment of diaphyseal fractures in an open fracture osteoporotic rat model
Source: Osteoporos Int. 2016 Nov 7;28(2):697–707. doi: 10.1007/s00198-016-3771-8 (PMC5269464; doi:10.1007/s00198-016-3771-8)
Supplement: Supplementary file 1 — (PDF 946 kb) [file 198_2016_3771_MOESM1_ESM.pdf]

*Supplementary Material:*

Representative callus cross-sections of each group obtained at a distance of 0.5 mm from fracture line.

*Article:*

Do osteoporotic fractures constitute a greater recalcitrant challenge for skeletal regeneration? Investigating the efficacy of BMP-7 and zoledronate treatment of diaphyseal fractures in an open fracture osteoporotic rat model.

*Journal:*

Osteoporosis International

*Authors:*

Neashan Mathavan
Magnus Tägil
Hanna Isaksson

**Corresponding Author:**

Neashan Mathavan, M.Sc.

Department of Biomedical Engineering

Lund University

PO Box 118, 221 00 Lund, SWEDEN

Phone: +46 46 222 06 59

Email: neashan.mathavan@bme.lth.se

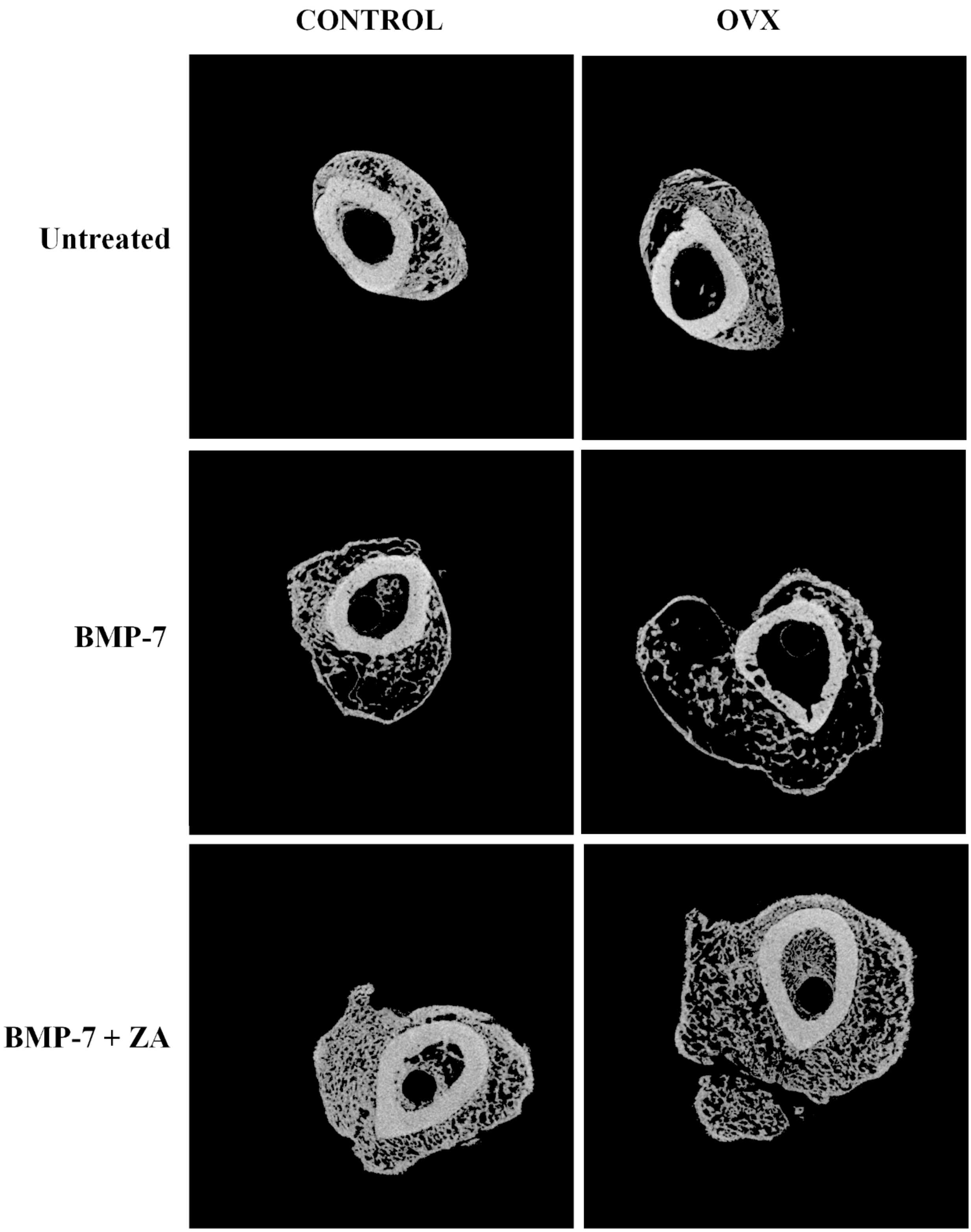


*Mathavan et al (2016) Osteoporos Int.*
